# Widespread Recruitment of ParA-like NTPases into Prokaryotic Immunity reveals a diversity of anti-selfish element strategies

**DOI:** 10.64898/2026.10.19.760177

**Published:** 2026-10-22

**Authors:** Huan Li, A. Maxwell Burroughs, L Aravind

## Abstract

The SIMIBI subgroup within the GTPase class of P-loop NTPases, including ParA/MinD, is best known for chromosome segregation, cell division, and macromolecular localization. Its role in immunity remains largely unexplored relative to TRAFAC-subgroup GTPases and other NTPases. Using systematic sequence, structure, and comparative genomics analysis, we surveyed the ParA clade for roles in immunity. ParA underwent at least two major, independent transitions from core cellular functions to prokaryotic immunity, producing two large "conflict" branches with diverse, uncharacterized systems, plus smaller, restricted immune lineages arising sporadically outside these branches. Across 28 conflict systems, ParA couples to a wide range of partners, including nuclease, NADase, and peptidase effectors, novel sensor domains such as a variant HeH domain, and apoptosis-inducing modules reminiscent of eukaryotic programmed cell death; several systems are enriched in multicellular bacteria, and a subset was transferred to eukaryotes on multiple occasions. Newly characterized ParA partner domains, including a nucleotide cyclase, a TIM-barrel domain, START, and permuted FHA domains, further define new anti-selfish-element systems independent of ParA. Together, these findings establish ParA as a recurrently co-opted "fulcrum" NTPase in prokaryotic immunity, predicted to act as an interceptor of invasive nucleic acids, a multimerizing effector, and a switch regulating other immune effectors.

